# The proliferative and multipotent epidermal progenitor cells for human skin reconstruction in vitro and in vivo

**DOI:** 10.1111/cpr.13284

**Published:** 2022-06-20

**Authors:** Jung Hwa Lim, Dae Hun Kim, Kyung Hee Noh, Cho‐Rok Jung, Hyun Mi Kang

**Affiliations:** ^1^ Korea Research Institute of Bioscience and Biotechnology (KRIBB) Daejeon Republic of Korea; ^2^ Department of Functional Genomics Korea University of Science and Technology (UST) Daejeon Republic of Korea

## Abstract

**Objectives:**

The skin exhibits tremendous regenerative potential, as different types of progenitor and stem cells regulate skin homeostasis and damage. However, in vitro primary keratinocytes present with several drawbacks, such as high donor variability, short lifespan, and limited donor tissue availability. Therefore, more stable primary keratinocytes are needed to generate multiple uniform in vitro and *in vivo* skin models.

**Results:**

We identified epidermal progenitor cells from primary keratinocytes using Integrin beta 1 (ITGB1) an epidermal stem cell marker markedly decreased after senescence in vitro. Epidermal progenitor cells exhibited unlimited proliferation and the potential for multipotent differentiation capacity. Moreover, they could completely differentiate to form an organotypic skin model including conversed mesenchymal cells in the dermis and could mimic the morphologic and biochemical processes of human epidermis. We also discovered that proliferation and the multipotent differentiation capacity of these cells relied on ITGB1 expression. Eventually, we examined the in vitro and *in vivo* wound healing capacity of these epidermal progenitor cells.

**Conclusions:**

Overall, the findings suggest that these stable and reproducible cells can differentiate into multiple lineages, including human skin models. They are a potentially powerful tool for studying skin regeneration, skin diseases, and are an alternative for *in vivo* experiments.

## Graphical abstract

Our stable and reproducible epidermal progenitor cells from human epidermis have proliferation and multipotent differentiation potentials, regeneration capacity and could generate *in vivo* mimic 3D skin model not only for regeneration therapy but also for alternative animal experiments.

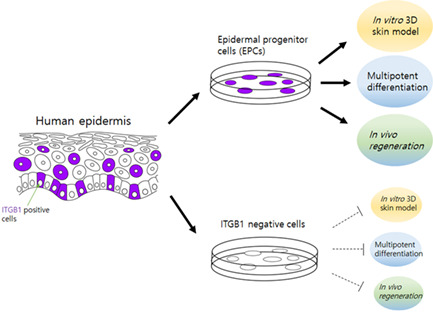

## 1 INTRODUCTION

The skin is the largest organ of the human body and is an important barrier that prevents pathogen entry; additionally, it demonstrates various functions, including immune functions and dehydration.^1^ Proliferation and migration of keratinocytes are critical for wound coverage and re‐epithelialization during wound healing.^2^ Moreover, the epidermis secretes various cytokines, proteases, and growth factors into the wound microenvironment to regulate the inflammatory response and to promote angiogenesis, wound closure, and extracellular matrix remodelling.^3^ The resident epidermal stem cells mediate the constant regeneration and repair of the human epidermis.^4^ Additionally, they generate other stem cells or transiently amplify progenitor cells that undergo terminal differentiation to form epidermal cells.^4^ The main sources of primary epidermal stem cells are neonatal foreskin or skin tissues obtained during skin biopsies. They are mainly located in the basal layer and hair follicle bulges that present a rich blood supply in the epidermis. Proliferation of these cells is regulated by growth factors and via establishment of intercellular contact.^3^, ^5^ Epidermal keratinocytes play a major role in modifying the wound microenvironment.^6^, ^7^ They secrete extracellular factors, such as growth factors, proteases, and extracellular proteins, that stimulate endothelial cells, fibroblasts, and myofibroblasts to facilitate wound healing, including angiogenesis and wound closing.^6^, ^7^ Studies have investigated epidermal keratinocytes for possible regenerative approaches since the 1970s. Most studies have demonstrated that these cells promote wound healing or replace lost skin by relying on their proliferation capacities.^8^ Integrins are the major cell surface receptors for adhesion of epidermal cells to both the basement membrane in the healthy skin and the provisional extracellular matrix in the wounded skin.^9^, ^10^ Several studies have reported that integrins are necessary for re‐epithelialization, cell migration, fibronectin assembly, and increased differentiation in the wounded epidermis.^11^ Moreover, several distinct markers that are distributed in the skin are used to identify and isolate specific populations of epidermal stem cells. Integrins, G protein‐coupled receptors containing leucine‐rich repeats, cytokeratins, transcription factors (such as sox9, gli1, and lhx2), cadherins, catenins, and p63 are known as epidermal stem cell biomarkers.^12^, ^13^, ^14^, ^15^, ^16^ However, only a few studies have evaluated all characteristics of epidermal stem cells, such as the potential to undergo multipotent differentiation.

Over the last decade, tissue‐engineered models that mimic human skin, known as human skin equivalents (HSEs), have been developed to replace *in vivo* animal models.^17^, ^18^ HSEs are 3D models of the human epidermis. They possess a multilayered epithelium composed of keratinocytes grown on a collagen substrate with dermal fibroblasts.^19^, ^20^, ^21^ HSEs are used in a diverse range of applications, including safety and risk assessments and biological efficacy. Particularly, in vitro experimentation using the HSEs is important because of the stringent laws that are applicable to animal experiments. Therefore, the HSEs that can efficiently mimic the skin epidermis using stable primary keratinocytes are required to replace in vivo experiments. Keratinocytes that are seeded on a dermal matrix in HSEs are brought to an air–liquid interface to form an epidermis that closely resembles that of the human skin.^22^ However, primary human keratinocytes present with several drawbacks, such as high donor variability, short in vitro lifespan, and limited donor tissue availability.^23^, ^24^ Recently, studies have established immortalized keratinocytes to overcome their short lifespans for the generation of multiple uniform HSEs from one donor cell line.^25^, ^26^ However, most available immortalized keratinocyte cell lines demonstrate no differentiation capacity, resulting in poor epidermal morphology and barrier function, or exhibit limited population doubling capacity.^27^, ^28^ Therefore, more stable primary keratinocytes are needed to generate multiple uniform *in vitro* HSEs to replace *in vivo* animal experiments.

Here, we established a stable epidermal progenitor cell population showing high proliferative capacity and the potential to undergo multipotent differentiation from human primary keratinocytes using *integrin beta 1 (ITGB1)*, an epidermal stem cell marker. They also successfully differentiated into a functional 3D model of the skin within 14 days of mimicking the skin epidermis and dermis *in vivo* and could converse into mesenchymal cells to replace mouse fibroblasts in the dermis supporting the maturation of epidermis. Remarkably, these cells could promote wound healing *in vitro* and *in vivo*, indicating that they were epidermal progenitor cells (EPCs), not transient amplifying cells, derived from the human epidermis.

## 2 MATERIALS AND METHODS

### 2.1 Keratinocyte culture and fluorescence cell sorting for epidermal progenitor cell isolation

Human primary keratinocytes were purchased from Biosolution (#HEK‐A/F, Seoul, Korea) and ATCC, and cultured in KGM‐gold medium in 0.2% gelatin‐coated culture plates at 5% CO_2_, at 37°C. For isolation of EPCs, cells were sorted using a FITC conjugated anti‐ITGB1 antibody. After 5–7 days, cells (at 80% confluence) were detached using 0.5% Trypsin–EDTA and passaged. The cumulative population doubling with each subculture was calculated with the formula 2X = NH/NI, where NI is the seeded cell number, NH is the cell harvest number at confluence (>80%), and X is population doubling. The calculated population doubling was then added to the previous population doubling level to yield the cumulative population doubling level.

### 2.2 Assessment of multi‐lineage differentiation potential

To induce adipogenic differentiation, cells (at passage 3) were cultured in an adipogenic medium consisting of RPMI‐1640, 10% FBS, 1 μM dexamethasone, 0.5 μM 3‐isobutyl‐1‐methylxanthine, 0.05 mg/L human insulin, and 60 μM indomethacin. After 14 days of culture, cells were stained using Oil red O for the presence of intracellular lipid droplets, indicative of adipocytes. To induce osteogenic differentiation, cells were cultured in RPMI‐1640, 10% FBS, 0.1 μM dexamethasone, 100 mM b‐glycerol phosphate, and 50 μM ascorbic acid‐2‐phosphate. After 14 days of culture, cells were stained using alizarin red staining indicating osteogenic differentiation. To induce chondrogenic differentiation, cells were cultured in a chondrogenic medium consisting of DMEM high glucose (DMEM‐HG), 0.1 μM dexamethasone, 50 μg/mL ascorbic acid‐2‐phosphate, 100 μg/mL sodium pyruvate, 40 μg/mL proline, 10 ng/mL transforming growth factor b3, 1 × ITS, and 1.25 mg/mL BSA. After 21 days of culture, cells were stained with Alcian blue to evaluate chondrogenic differentiation.

### 2.3 Human skin equivalents

The irradiated 3 T3 cells or mesenchymal cells (3 × 10^5^ cells/24 well multi insert dish) were seeded onto matrix consisting of bovine collagen type I, Matrigel, and 5 μg/mL of fibronectin. These fibroblast sheets were cultured submerged for 1 week in the DMEM‐HG supplemented with 10% FBS. The primary cells, EPCs or EPCs transduced shCTL or shITGB1 lentivirus (1 × 10^6^ cells/24 well multi insert dish) were seeded onto the fibroblast sheets. After 6 h of attachment, keratinocyte culture medium (KGM‐gold) was added the cells for 3–4 days and then cells were placed air‐exposed for 7–10 days in DMEM/F12 supplemented with 2% FBS, 1 μM hydrocortisone, 1 × ITS and 1.8 mM CaCl_2_. Medium was changed every day.

### 2.4 In vivo wound healing assay

Seven‐week‐old female BALB/c nude mice (BALB/cSlc‐nu/nu) were purchased from Japan SLC, Inc. All experimental protocols were approved by the Korea Research Institute of Bioscience and Biotechnology (KRIBB) Animal Care and Uses Committee. All of procedures were performed by accordance with the appropriate KRIBB biosafety guidelines and regulations. Mice were anaesthetized with 2% Avertin solution (0.1 ml/20 g body weight; Bayer, Leverkusen, Germany). The back of the mouse was sterilized using an alcohol swab and a sterile biopsy punch (6‐mm diameter) was used for making two wounds in their dorsal skin. Mice were randomized into the following four groups: a control group, which received 50 μl of 1 × 10^6^/each site unsorted cells at passage 5, EPCs group, which received 50 μl of 1 × 10^6^/each site EPCs, EPCs‐shCTL group, which received 50 μl of 1 × 10^6^/each site EPCs transduced the shCTL lentivirus, and EPCs‐shITGB1 group, which received 50 μl of 1 × 10^6^/each site EPCs transduced the shITGB1 lentivirus. The wound area was measured every day for 10 days after wounding. On the tenth day, mice were sacrificed, wound skin samples were collected, fixed in 4% paraformaldehyde, embedded, and sectioned for morphometric analysis and immunohistochemistry.

### 2.5 Statistical analyses

The data are presented as means ± SEM. Unpaired Student's *t* test was used for comparisons between two groups. We used one‐way ANOVA with Tukey's post hoc tests to compare multiple groups. A *p* value <0.05 was considered to be significant.

### 2.6 Supplementary materials and methods

Antibodies and reagents, colony‐forming unit assay, quantitative real‐time PCR, immunofluorescence and immunocytochemical analysis, flow cytometry, skin irritant test, scratch wound closure assay, tube formation assay, angiogenesis antibody arrays are described in supplementary materials and methods.

## 3 RESULTS

### 3.1 ITGB1‐positive cells exhibit epidermal progenitor cell properties *in vitro*

**FIGURE 1:**
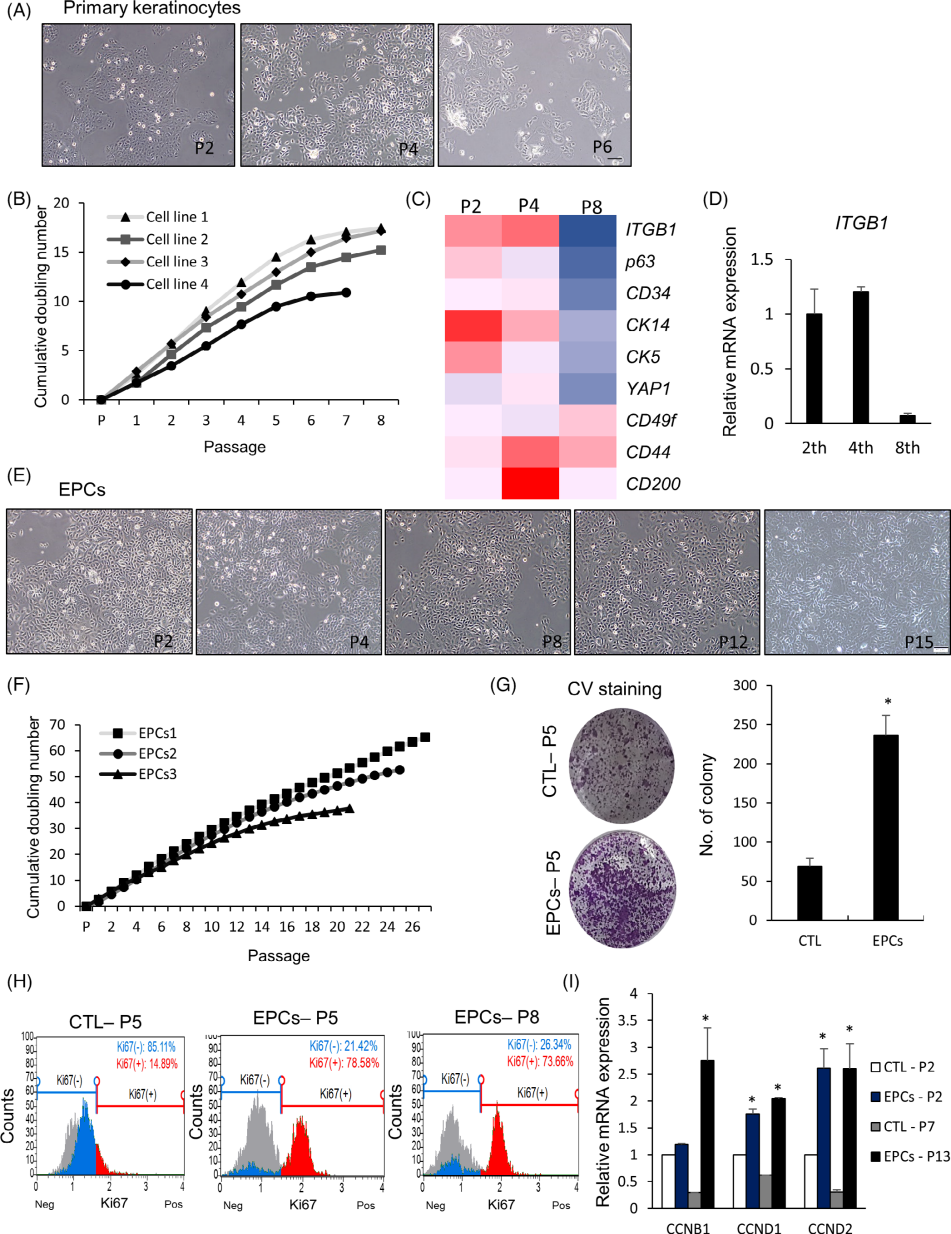
Establishment and characterization of epidermal progenitor cells from human primary keratinocytes. (A) Morphology of human primary keratinocytes in vitro. (B) Cumulative population doubling numbers for four primary keratinocyte cell lines throughout ex vivo expansion. (C) In vitro gene expression patterns of epidermal stem cell markers in human primary keratinocytes. (D) Quantitative RT‐PCR analysis of integrin beta 1 (ITGB1) expression in human primary keratinocytes at different passage. (E) Morphology of human epidermal progenitor cells (EPCs) from passage 2 to passage 15. (F) Cumulative population doubling numbers for three EPCs cell lines throughout ex vivo expansion. (G) Representative images of crystal violet stained colonies of EPCs and CTL cells at passage 5. (H) FACS analysis of cell proliferative potential of EPCs at passage 5 and 8 and CTL cells at passage 5. (I) Quantitative RT‐PCR analysis of proliferation‐related markers in EPCs and CTL cells at passages 2, 7, and 13. All data are depicted as mean ± SEM. *p < 0.05 compared to CTL cells via unpaired Student's *t*‐test. Scale bar, 50 μm

First, we maintained human primary keratinocytes purchase from Biosolution (#HEK‐A/F, Seoul, Korea) and ATCC in vitro to characterize their proliferation properties. Cells were homogenous until passage 4, but lost their morphology and presented a decreased growth rate after passage 6 (Figure 1A,B). To identify the proliferation‐associated stem cell markers in the keratinocytes, we examined the expression of epidermal stem cell markers at different passages (Figure 1C). We observed that the expression of *ITGB1, p63, CD34, CK14*, *CK5*, and *yes‐associated protein 1 (YAP1)* was substantially decreased in the primary keratinocytes after passage 8; the cells also demonstrated a decreased in vitro growth rate during this passage. However, expression of *CD44, CD49f*, and *CD200* did not change through the passages (Figure 1C). We also observed primary keratinocyte at passage 3 expressed not only ITGB1 but also other skin stem cell markers such as CK14, p63, and YAP1 (Figure S1A). Since *ITGB1* expression presented with the most significant decrease with increase in passage number (Figure 1D), we hypothesized that *ITGB1* expression was related to the proliferation of human primary keratinocytes. Different 3 cell lines were used for sorting the *ITGB1* positive cells, and average positive percentages of total cells were 36.7% (Figure S1B). Next, we examined whether ITGB1‐positive cells possessed progenitor cell properties *in vitro*. We found that these cells maintained their morphology and growth rate throughout the culture period (Figure 1E). Moreover, they underwent an average of 51.8 population doublings, indicating that these cells possessed a higher proliferation rate (Figure 1F) than unsorted primary keratinocytes (Figure 1B). After two weeks of culture, ITGB1‐positive cells exhibited a high clonal expansion capacity with extremely low cell density compared to the unsorted cells at passage 5 (Figure 1G). While above 70% of ITGB1‐positive cells continuously expressed markers associated with cell proliferation, such as Ki67, until passage 8, only 15% of the unsorted cells were proliferative at passage 5 (Figure 1H). Moreover, ITGB1‐positive cells demonstrated a higher expression of cyclins than unsorted primary keratinocytes and exhibited consistent expression till passage 13 (Figure 1I). Immunostaining and FACS analysis revealed that ITGB1‐positive cells also expressed skin stem cell markers, such as CK14, YAP1, and p63 (Figure S2A,B). Moreover, some ITGB1 positive cells also co‐expressed at least one of other stem cell marker (Figure S2C). We also examined the profiles of surface markers of these cells and compared them with those of adipose tissue‐derived mesenchymal stem cells. Notably, the ITGB1‐positive cells expressed only CD44 and CD166 mesenchymal stem cell markers but unsorted CTL cells expressed these markers weakly nor not at all (Figure S3). Therefore, our findings demonstrated that ITGB1‐positive cells were distinct epidermal progenitor cells (EPCs) that possessed a high proliferation rate *in vitro*.

### 3.2 ITGB1‐positive EPCs possess the potential to undergo multipotent differentiation *in vitro*

**FIGURE 2:**
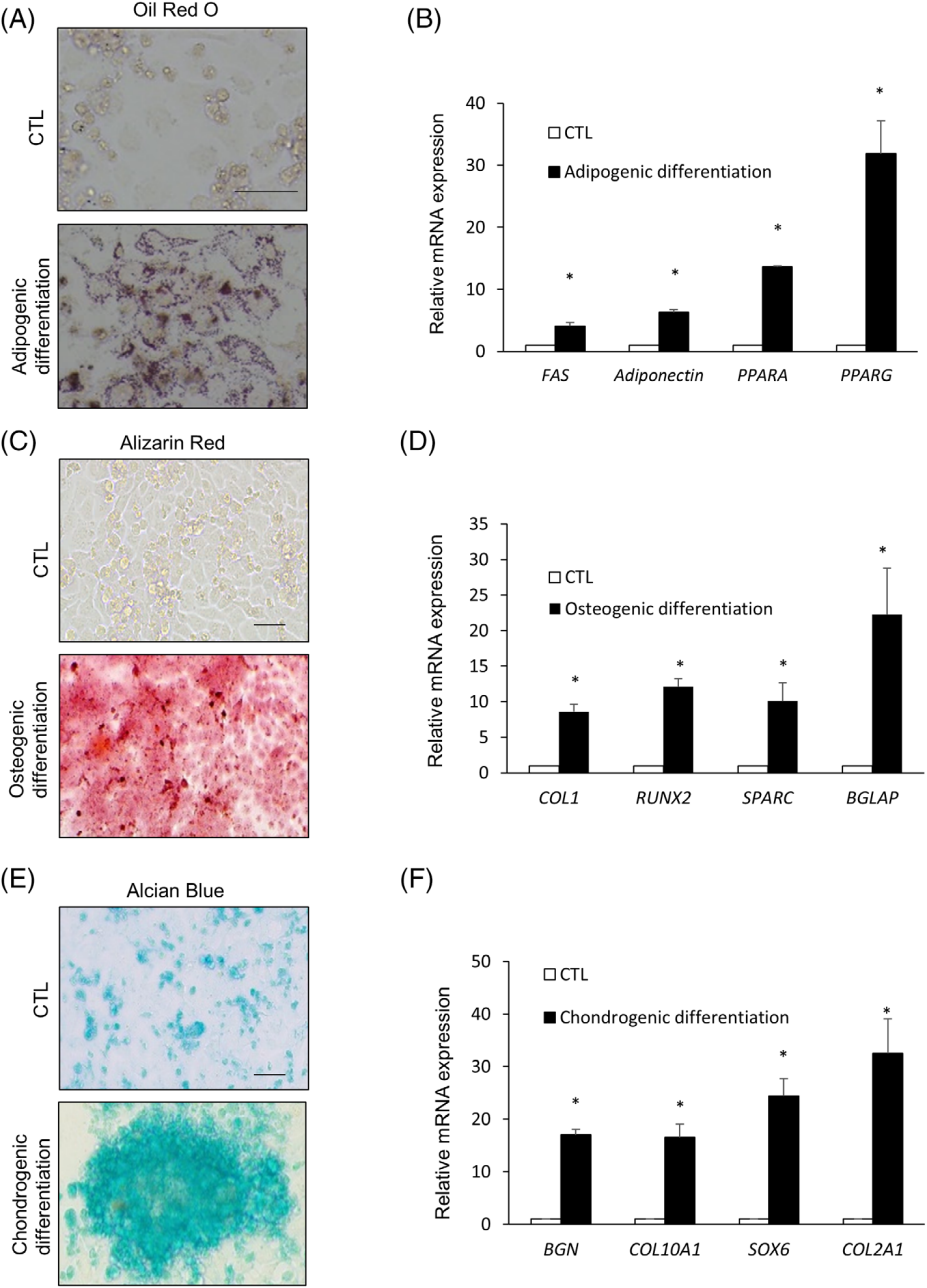
Multipotent differentiation potential of EPCs in vitro. (A) Representative image of adipogenic differentiation of EPCs assessed via Oil Red O staining. (B) Transcript levels of adipocyte markers. (C) Representative image of osteogenic differentiation of EPCs assessed via alizarin red staining. (D) Transcript levels of osteoblast markers. (E) Representative image of chondrogenic differentiation of EPCs assessed via Alcian blue staining. (F) Transcript levels of chondrocyte markers. All data are shown as the mean ± SEM. *p < 0.05, compared to CTL via unpaired Student's *t*‐test. Scale bar, 200 μm

Previous studies have demonstrated that rodent and human skin‐derived progenitor cells can differentiate into both neuronal and mesodermal cell types.^29^, ^30^ We examined the potential of these cells to undergo multipotent differentiation into mesodermal cell lineages. EPCs cultured in an adipogenic medium for two weeks exhibited intense cytoplasmic staining for Oil Red O at passage 5, indicating lipid accumulation (Figure 2A) and adipocyte‐related gene expression (Figure 2B). Post two weeks of culturing in an osteogenic medium, EPCs at passage 5 exhibited positive staining for alizarin (Figure 2C), an osteogenic stain. They also expressed osteogenic markers, such as *COL1A*, *RUNX2*, and *BGLAP* (Figure 2D). Additionally, at passage 5, EPCs cultured in chondrogenic medium for three weeks displayed chondrocyte‐specific Alcian blue staining (Figure 2E) and expressed high levels of chondrogenic differentiation markers (Figure 2F). These data suggested that EPCs possessed the potential to differentiate into cells of all three germ layers.

### 3.3 Construction of a 3D human skin equivalents (HSEs)  using EPCs

**FIGURE 3:**
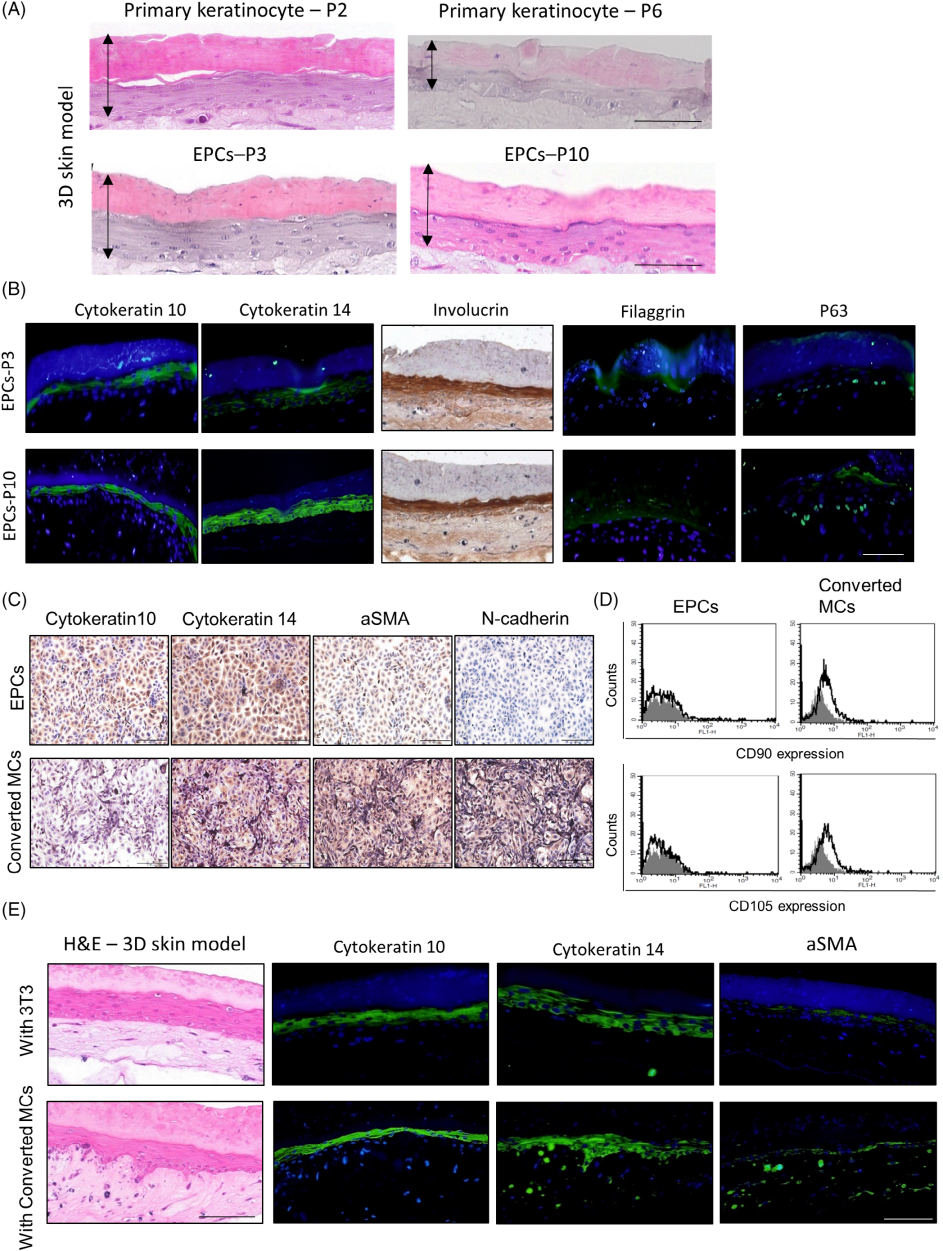
EPCs reconstructed skin equivalent models mimic epidermis with mesenchymal cells in the dermis layer in vitro. (A) Representative images depicting H&E staining results of 3D skin models generated by using primary keratinocytes (passages 2 and 6) and epidermal progenitor cells (EPCs) at early and late passages (passages 3 and 10). Arrow indicates the thickness of the epidermis in the skin equivalent model. (B) Immunostaining of skin epidermis markers in EPCs‐derived skin equivalent models at passages 3 and 10. (C) Expression of epidermal to mesenchymal transition (EMT) markers post transition of EPCs to mesenchymal cells (MCs). (D) Expression of mesenchymal stem cell markers, such as CD90 and CD105, in EPCs and transition of EPCs to mesenchymal cells (MCs). (E) Representative immunostaining image of EPCs‐derived skin equivalent models supported by the dermis, consisting of mitomycin‐treated 3 T3 cells or mesenchymal cells (MCs) derived from EPCs. Scale bar, 100 μm

We examined the ability of EPCs to differentiate into a 3D skin model similar to that of unsorted cells at the early passage (passage 2). We observed that the EPCs at early and late passages (passages 3 and 10, respectively) could differentiate into a 3D human epidermal skin model. This model demonstrated the presence of human epidermis‐like structures, such as a multilayered basal layer, stratum spinosum, granular layer, and stratum corneum (Figure 3A), similar to those formed by unsorted primary skin cells at early passage (at passage 2, Figure 3A). Immunohistochemical staining revealed the expression patterns of markers in the epidermal layers of the 3D model similar to those of the skin epidermis, such as the expression of cytokeratin 10 in the supra‐basal layer, cytokeratin 14 in the basal layer, and involucrin and filaggrin in the granular layer. Moreover, p63 expression in basal cells was detected in the 3D model at a later passage (passage 10), indicating that epidermal keratinocyte stemness was maintained in the EPCs at later passages (Figure 3B). Furthermore, we attempted to induce the transition of EPCs to mesenchymal cells to replace irradiated 3 T3 cells in the dermis and to generate whole HSEs using EPCs. Mesenchymal cells exhibited an increased expression of mesenchymal markers, such as α‐SMA and N‐cadherin, and a decreased expression of mature epithelial cell markers, such as cytokeratin 10 (Figure 3C). Expression of the epidermal stem cell marker, cytokeratin 14, did not differ post transition. Moreover, some of these mesenchymal cells expressed mesenchymal stem cell markers, such as CD90 and CD105 (Figure 3D). Subsequently, we examined whether these cells could replace irradiated 3 T3 cells in the dermis of the 3D skin model. The 3D skin model with conversed mesenchymal cells possessed a more mature human epidermis‐like structure with a dermis layer than those of generated with irradiated 3 T3 cells (Figure 3E). Moreover, the cells in the dermis layer expressed α‐SMA (Figure 3E). We also investigated whether the models generated by EPCs in the later passages (passage 10) could be used to conduct skin irritation tests in vitro. Non‐irritant and irritant chemicals were selected from the list in the performance standards of OECD TG439,^31^ and PBS and 5% SDS were used as negative and positive controls, respectively. Treatment with diethyl phthalate, a non‐irritant, produced unremarkable changes in the morphology and cell viability of the 3D model. However, most epidermal cells were swollen, and more than 70% cell death was observed 24 h after treatment with tetrachloroethylene, a skin irritant (Figure S4A,B). These results suggested that EPCs formed reproducible stable cell lines that mimicked the skin epidermis in HSEs and could be used as potential alternatives for *in vivo* studies. Moreover, these cells transitioned into mesenchymal cells to replace non‐human fibroblasts in the dermis to support the maturation of the epidermis in the HSEs.

### 3.4 ITGB1 is a key regulator in the proliferation and differentiation of EPCs

**FIGURE 4:**
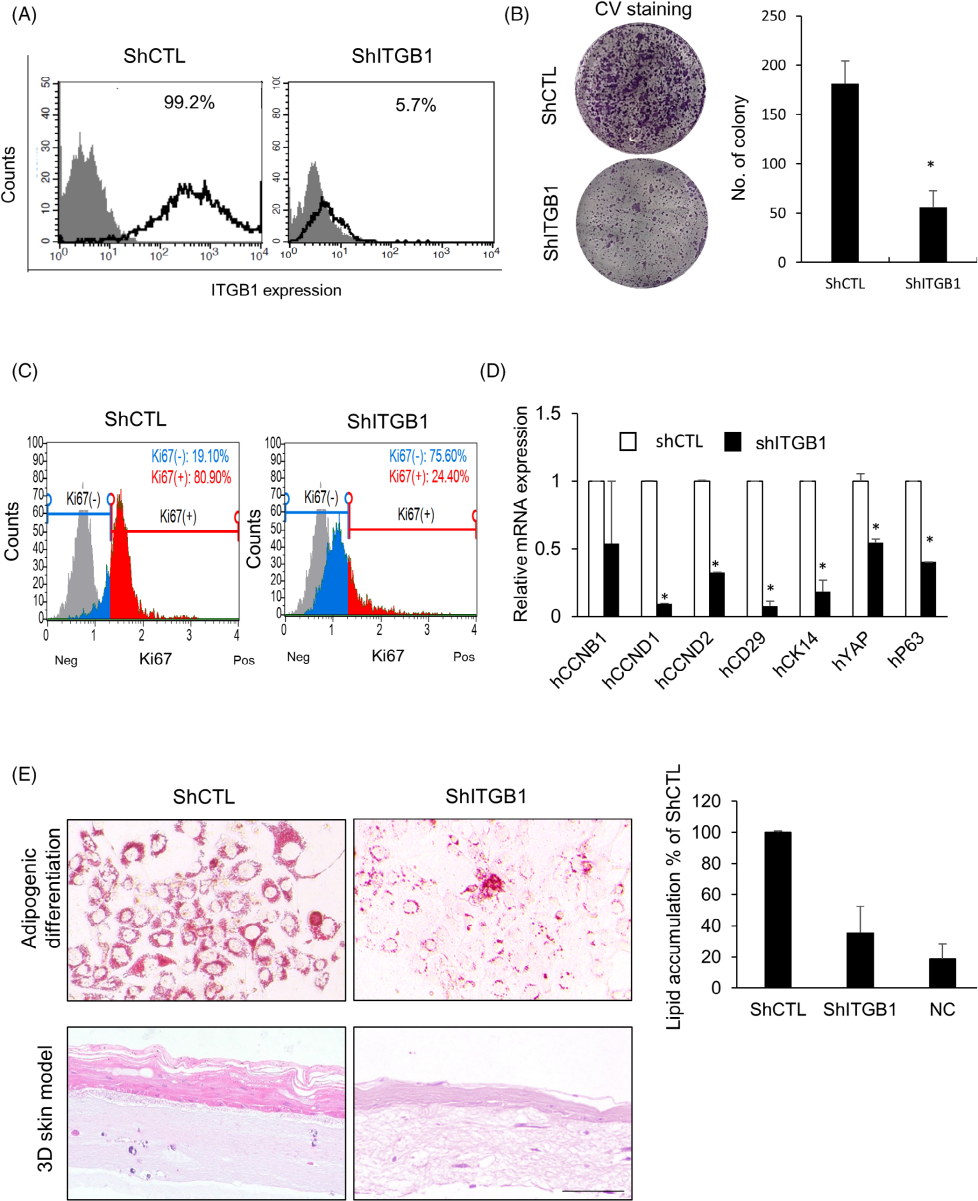
Effect of ITGB1 knockdown on the proliferation and differentiation of epidermal progenitor cells. (A) ITGB1 expression post knockdown by lentiviral shRNA. (B) Representative images of crystal violet stained colonies of lentiviral shCTL and shITGB1 cells derived from EPCs. (C) FACS analysis of proliferative EPCs subjected to transduction with shCTL and shITGB1. (D) Quantitative RT‐PCR analysis of proliferation and epidermal stem cell‐related markers in cells subjected to transduction with shCTL and shITGB1. (E) Multipotent differentiation potential of EPCs subjected to transduction with shCTL and shITGB1. Representative images of Oil red O staining and quantitative analysis of Oil red O performed for ascertaining adipogenic differentiation, and skin equivalent models generated by using EPCs subjected to transduction with shCTL and shITGB1. All data are shown as mean ± SEM. *p < 0.05 compared to the control via unpaired Student's *t*‐test. Scale bar, 200 μm

To investigate the precise role of ITGB1 in the proliferation and differentiation of primary keratinocytes, ITGB1 was subjected to knockdown using lentiviral shRNA and siRNA, or using a specific antibody and we examined the proliferation rate and differentiation capacities of EPCs. We also evaluated the expression levels of proliferation‐related markers and skin stem cell markers (Figure 4 and Figure S5). We observed that *ITGB1* expression levels decreased in cells subjected to transduction with lentiviral shITGB1 (Figure 4A). This decrease reduced the clonal expansion capacity of the shITGB1‐transduced cells after two weeks of culture and substantially reduced their cell density compared to the shCTL‐transduced cells (Figure 4B). Low ITGB1 expression was also associated with decreased Ki67‐positive cell population (Figure 4C). Moreover, qRT‐PCR analysis demonstrated that ITGB1 knockdown resulted in the decreased expression of proliferation‐related markers, such as *cyclin D1* and *cyclin D2*, and skin stem cell markers, such as *CK14, YAP* and *p63* (Figure 4D). We recorded similar observations in cells subjected to treatment with ITGB1 siRNA (Figure S5A‐C) and anti‐ITGB1 blocking antibody (Figure S5D).

We also examined the effects of ITGB1 expression on the multipotent differentiation potential of the EPCs. EPCs, after treatment with lentiviral shCTL and shITGB1, were cultured in adipogenic media for two to three weeks. We observed that accumulation of lipid droplets substantially decreased in the differentiated cells after ITGB1 knockdown compared to that in the shCTL‐treated cells (Figure 4E). Similar results were observed in the 3D HSEs (Figure 4E). Thus, these results suggested that the proliferation and multipotent differentiation capacity of the EPCs relied on ITGB1 expression.

### 3.5 EPCs display properties that aid wound healing and skin regeneration *in vitro* and *in vivo*

**FIGURE 5:**
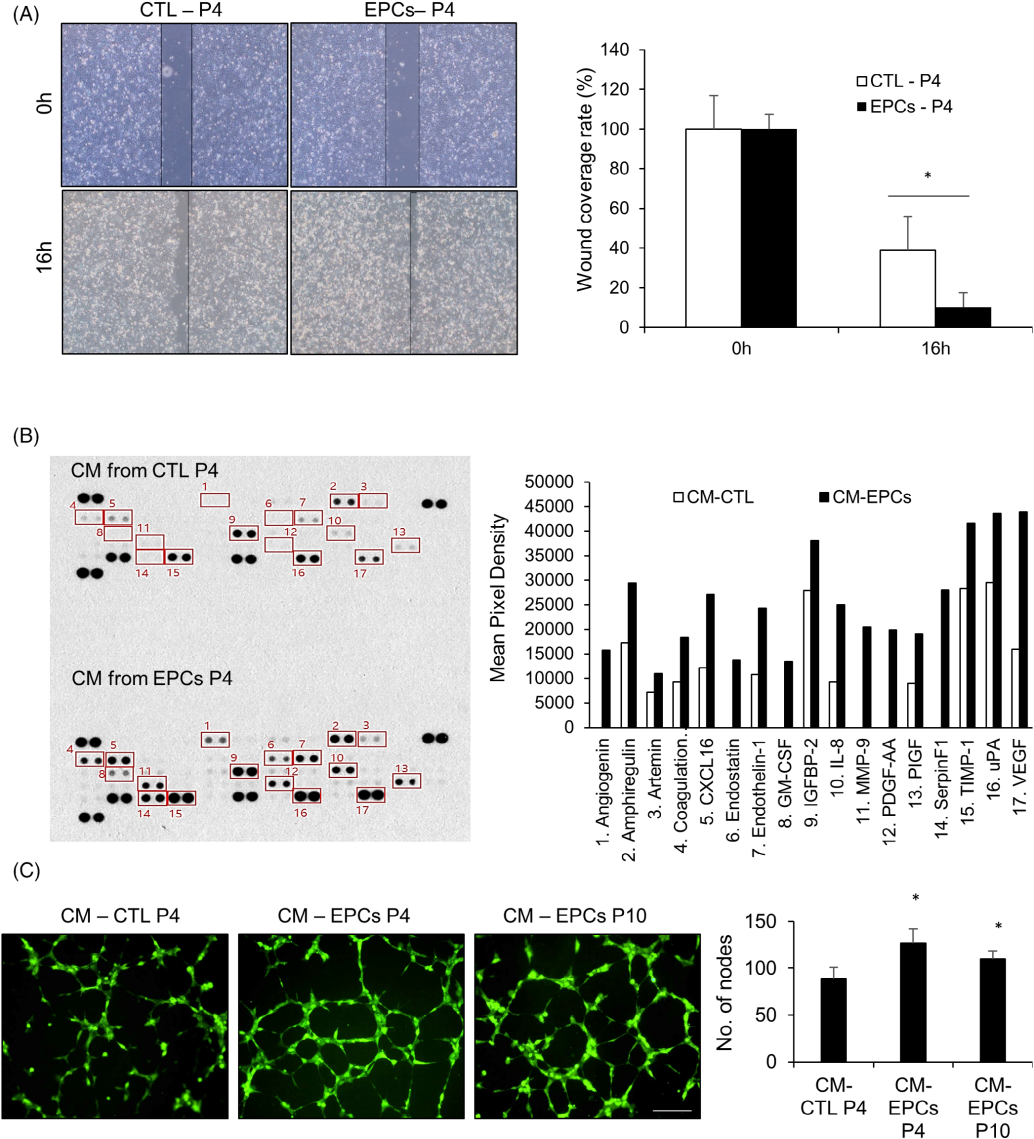
Molecular and wound healing assessment of epidermal progenitor cells in vitro. (A) Representative images of scratch wound covering with CTL and epidermal progenitor cells (EPCs; left), and wound coverage rate (%, right) at 0 and 16 h. (B) Angiogenic profile of the conditioned medium derived from CTL cells and EPCs. Labelled spot corresponds to an angiogenic factor shown in the graph on the right. (C) Tube formation assays for human umbilical vein endothelial cells cultured in the conditioned medium derived from EPCs and CTL cells. All data are depicted as mean ± SEM. *p < 0.05 compared to the control via unpaired Student's *t*‐test. Scale bar, 200 μm

To examine the regeneration capacity of EPCs *in vitro*, we performed a scratch wound assay using unsorted CTL cells and EPCs. The unhealed area percentage was smaller at 16 h post creation of a wound in the EPCs than that in the CTL cells (Figure 5A). Subsequently, we prepared a conditioned medium (CM) derived from both these cell types to investigate the role of their paracrine activities in immune cell migration. Moreover, we analysed the cytokine array data to identify the cytokines involved in in vitro skin regeneration. We found that the levels of various cytokines related to angiogenesis, including CXCL16, GM‐SCF, and VEGF, increased in the cells subjected to treatment with EPCs‐derived CM than those subjected to treatment with CTL‐derived CM cells (Figure 5B). Eventually, we examined the angiogenic activity of the cells subjected to treatment with EPCs‐derived CM. Human umbilical vein endothelial cells were cultured with CTL‐ and EPCs‐derived CM at passage 4 or at passage 10 for 6 h. The number of nodes per field of view was higher in the cells subjected to treatment with EPCs‐derived CM than that in cells subjected to treatment with CTL‐derived CM (Figure 5C). These results indicated that EPCs secreted key factors that promoted *in vitro* wound healing, and angiogenesis.

**FIGURE 6:**
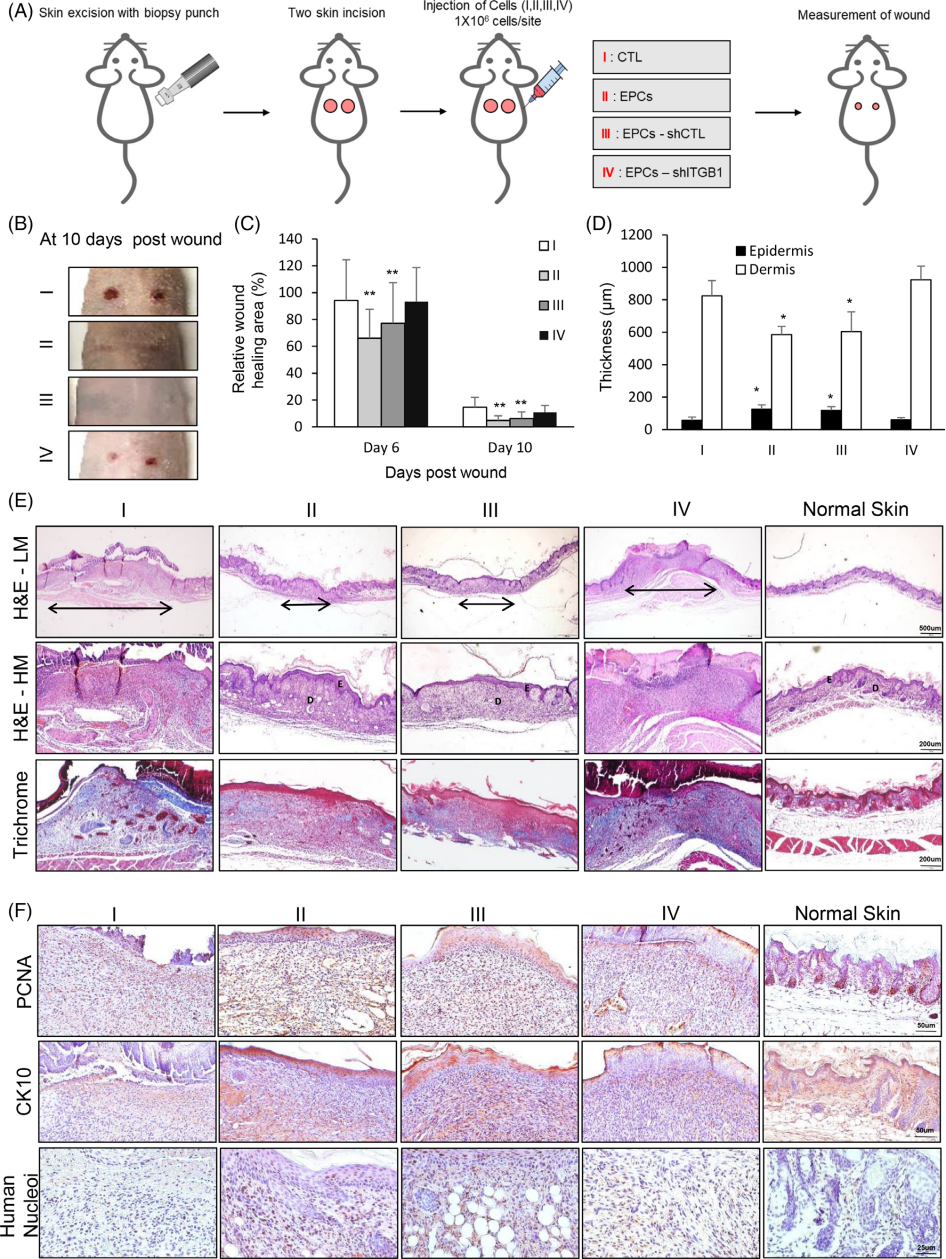
Skin wound healing effects of epidermal progenitor cells in vivo. (A) Schematic representation of the in vivo transplantation approach. Mice were randomly divided into four groups: CTL cell‐injected (I), epidermal progenitor cell (EPCs)‐injected (II), EPCs‐transduced shCTL‐injected (III) and EPCs‐transduced shITGB1‐injected (IV). (B) Mice skin at 10 days post wounding. (C) Area percentage of wound healing area in mice injected with different cells at day 6 and 10 post wounding. (D) Thickness of the epidermis and dermis in mice injected with different cells 10 days post wounding. (E) Representative images of H&E staining with low (upper, LM) and high magnification (middle, HM) and trichrome staining (lower panel) of mice injected with different cells. Arrow indicates the wound area of each group. (F) Immunostaining with the proliferation marker (PCNA), mature epidermis marker (CK10), and human cell marker (human nuclei) in mice injected with different cells. Normal skin tissues were used as a positive control. All data are shown as mean ± SEM. *p < 0.05 compared to the control via unpaired Student's *t*‐test. Scale bar, 200 μm

Thereafter, we determined the effects of the EPCs on wound healing of the skin *in vivo*. We used an excisional wound model to observe the healing process in nude mice (Figure 6A). The wound closure percentage was significantly higher in the EPCs‐treated group than that in the control group on days 6 and 10 after wound induction (Figure 6B,C and Figure S6A). To provide additional evidence that ITGB1 expression was linked specifically to skin regeneration and wound healing, ITGB1 was subjected to knockdown in EPCs using the lentiviral shITGB1. Subsequently, we compared the effects of this knockdown on wound healing with those triggered by EPCs subjected to transduction with the empty lentiviral vector shCTL (Figure 6A). The relative wound healing area was significantly greater in mice injected with shCTL EPCs than that in mice injected with shITGB1 EPCs (Figure 6C and Figure S6C). Furthermore, the wounded tissue samples subjected to treatment with ITGB1‐positive EPCs or shCTL EPCs exhibited almost complete healing of the epidermis and demonstrated similar collagen density and muscle healing as that of the normal skin. However, those subjected to treatment with CTL or shITGB1 EPCs exhibited a thin epidermal tissue, a high collagen density, and a damaged muscle layer (Figure 6D,E). Immunostaining analysis revealed that healed skin tissue, post injection with ITGB1‐positive EPCs, possessed increased number of PCNA‐positive cells in the epidermis and dermis compared to healed skin tissue treated with ITGB1‐negative EPCs (Figure 6F). Moreover, CD10 staining results concurred with these findings (Figure 6F). Interestingly, the skin epidermis healed with ITGB1‐positive EPCs‐based treatment presented with a significantly increased human nucleoi‐positive cells compared to the skin epidermis of mice injected with ITGB1‐negative EPCs, indicating that the EPCs retained their proliferation properties in vivo post transplantation (Figure 6F). Moreover, to further examine the functions of repaired skin, immunostaining for epidermal tight junctions, polarity, dermal laminin structure, and immune cells were performed. CK14 expression observed in the basal layer of multilayer epidermis indicated normal polarity in the epidermal cells. Tight junction protein was also strongly expressed in the keratinocytes in the epidermis, and laminin 5 expression was observed in the basement membrane between epidermis and dermis in the repaired skin treated with ITGB1‐positive EPCs or shCTL EPCs similar to normal skin (Figure S7). In addition, proinflammatory macrophage/monocytes staining for CD11b also dramatically decreased in the wounded tissue samples treated with ITGB1‐positive EPCs or shCTL EPCs compared to those with crude or shITGB1 cells (Figure S7). Collectively, these observations indicated that EPCs promoted wound healing and epidermal regeneration *in vitro* and *in vivo*.

## 4 DISCUSSION

In this study, we aimed to identify and characterize ITGB1‐positive EPCs obtained from the human epidermis. These cells are proliferative and possess the ability to undergo multipotent differentiation into three different cell types. Moreover, these cells promote wound healing in vitro and in vivo via re‐epithelialization, including migration and proliferation of cells and secretion of various cytokines.

Several different integrins play important roles in wounded keratinocytes, such as re‐epithelialization that includes cell proliferation and migration and extracellular matrix assembly.^32^ Integrins play diverse roles in several biological processes, including developmental cell migration, wound healing, cell differentiation, and apoptosis. Particularly, the anchorage and interaction of epidermal keratinocytes with the basement membrane are largely mediated by integrins.^33^ They are adhesive proteins that can sense various aspects of the environment. When epidermal morphogenesis proceeds, integrin patterns and bone marrow organization achieve a normal state that is tightly correlated with tissue homeostasis.^34^ Studies have reported a high expression of beta‐1 and alpha‐6 integrins is a marker of human epidermal stem cells and is associated with the maintenance of the epidermal stem cell functions.^35^, ^36^ The beta‐1 integrin ITGB1 has been reported to regulate the differentiation and proliferation of keratinocytes and is a putative stem cell marker in the interfollicular epidermis.^36^, ^37^, ^38^ In this study, we identified that ITGB1 is a strong marker of EPCs proliferation and differentiation. Moreover, we demonstrated that ITGB1 knockdown in EPCs reduced the proliferation, differentiation, and *in vivo* wound healing capacity of these cells. Data obtained by the Zhang group showed that YAP was highly expressed in progenitors of the single‐layered basal epidermis.^39^ The Laurikkala group reported that p63, a member of the p53 family of proto‐oncogenes, was necessary for the basal layer of the skin to maintain its proliferative capacity.^17^ Interestingly, our observations revealed that ITGB1‐positive cells demonstrated a high YAP and p63 expression. These results indicate a potential overlap between these populations or highlight an interconversion between different stem cell compartments in the skin epidermal cells.

Our study presents with several important implications for HSE models. Over the last two decades, the developed HSE models have reflected a considerable proportion of biological processes in the human epidermis in vivo. However, these models present with two major limitations. First, to establish primary keratinocyte‐derived HSEs, skin biopsies are necessary but are a huge burden for patients. Second, primary keratinocytes exhibit a limited life span and approximately 15 population doublings, allowing only a limited number of experiments to be performed using cells obtained from one donor. Therefore, in this study, we established a new stable primary keratinocyte cell line that exhibited epidermal stem cell‐like characteristics. Importantly, these cell lines possess the ability to proliferate and differentiate into HSE models using cells from early and late passages. Furthermore, these 3D skin models mimic the human epidermis structurally and functionally and are applicable as alternatives for *in vivo* experiments. In summary, we identified EPCs derived from primary keratinocytes that could differentiate into three different cell types, also form HSE models that mimicked the human epidermis *in vivo* and possessed wound healing abilities. Thus, these cells may be considered a powerful new tool for studying skin regeneration, wound healing, and various skin diseases and are an alternative for *in vivo* experiments.

## AUTHOR CONTRIBUTIONS

Conceived and designed the experiments: HMK, and CRJ. Performed the experiments and analysed the data: HMK, JHL, DHK, and KHN. Contributed reagents and materials: DHK. Discussed the results and commented on the manuscript: HMK, and CRJ. Drafted and revised the paper: HMK, and CRJ.

## CONFLICT OF INTERESTS

The authors declare that they have no competing interests.

## Supporting information


**Appendix S1** **Figure 1** Expression of integrin beta 1 profiles in the primary keratinocytes. (A) FACS analysis for skin stem cell marker of primary keratinocytes at passage 3. (B) FACS analysis and sorting the integrin beta 1 (ITGB1) expressing cells in the three different primary keratinocytes at passage 3
**Figure 2** Expression of skin stem cell markers of epidermal progenitor cells. (A) Representative immunostaining images of skin stem cell marker expression of primary keratinocyte (CTL) or epidermal progenitor cells (EPCs) at passage 5. (B) and (C). FACS analysis for skin stem cell marker of EPCs at passage 5. Scale bar, 50 μm
**Figure 3** Expression of mesenchymal stem cell markers of epidermal progenitor cells. Representative FACS analysis results of mesenchymal stem cell markers of primary keratinocyte (CTL), or epidermal progenitor cells (EPCs) at passage 4. Human adipose mesenchymal stem cell (MSC) were used as positive control
**Figure 4** Skin irritation test using skin equivalents model generated by epidermal progenitor cells. (A) Representative images depicting H&E staining results of 3D skin models generated by using epidermal progenitor like (EPCs) after treated non‐irritant (Diethylphthalate) and irritant (Tetrachloroethylene) chemicals. 5% SDS was used as positive control for irritant chemical. (B) Cell viability of cell from 3D skin models treated non‐irritant and irritant chemicals. All data are shown as the mean ± SEM. *p < 0.05, compared to CTL via unpaired Student’s *t*‐test. Scale bar, 200 μm
**Figure 5** Effect of integrin beta 1 knockdown on the proliferation of epidermal progenitor cells. (A) FACS analysis of proliferative EPCs subjected to transfection with siCTL and siITGB1 at 300 pM or 1 nM of concentration, respectively. (B) Quantitative RT‐PCR analysis of proliferation and epidermal stem cell‐related markers in cells subjected to transduction with siCTL and siITGB1 at 300 pM or 1 nM of concentration, respectively. (C) Western blotting analysis for proliferation and epidermal stem cell‐related markers in cells subjected to transduction with siCTL and siITGB1 at 300 pM or 1 nM of concentration, respectively. (D) FACS analysis of proliferative EPCs subjected to treated with PBS (CTL) and ITGB1 blocking antibody at 1 μg/ml or 5 μg/ml of concentration, respectively
**Figure 6** In vivo wound healing effects of epidermal progenitor cells. (A) Mice skin at 10 days post wound. Mice were randomly divided into four groups; CTL cell‐injected, epidermal progenitor cell (EPCs)‐injected, EPCs‐transduced shCTL‐injected and EPCs‐transduced shITGB1‐injected. (B) Area percentage of wound healing area in mouse subjected to injection using different cells for 10 days post wound. All data are shown as the mean ± SEM. *p<0.05, compared to CTL via unpaired Student’s *t*‐test
**Figure 7** In vivo function analysis of repaired skin treated with epidermal progenitor cells. Immunostaining for the epidermal polarity (CK14), the barrier function (tight junction protein‐1, TJP1) of the epidermis, a major link between the epidermis and dermis (laminin 5), and pro‐inflammation response (CD11b) in the dermis of the mice injected with different cells: CTL cell‐injected (I), epidermal progenitor cell (EPCs)‐injected (II), EPCs‐transduced shCTL‐injected (III) and EPCs‐transduced shITGB1‐injected (IV). Normal skin tissues were used as a positive control. Scale bar, 50 μm
**Table I** Sequences of oligonucleotide primers used for qPCR

## Data Availability

Data sharing is not applicable to this article as no new data were created or analyzed in this study.
