# Supplementary material for: The proliferative and multipotent epidermal progenitor cells for human skin reconstruction in vitro and in vivo
Source: Cell Prolif. 2022 Jun 20;55(9):e13284. doi: 10.1111/cpr.13284 (PMC9436902; doi:10.1111/cpr.13284)
Supplement: Supplementary file 1 — Appendix S1 Figure 1 Expression of integrin beta 1 profiles in the primary keratinocytes. (A) FACS analysis for skin stem cell marker of primary keratinocytes at passage 3. (B) FACS analysis and sorting the integrin beta 1 (ITGB1) expressing cells in the three different primary keratinocytes at passage 3 Figure 2 Expression of skin stem cell markers of epidermal progenitor cells. (A) Representative immunostaining images of skin stem cell marker expression of primary keratinocyte (CTL) or epidermal progenitor cells (EPCs) at passage 5. (B) and (C). FACS analysis for skin stem cell marker of EPCs at passage 5. Scale bar, 50 μm Figure 3 Expression of mesenchymal stem cell markers of epidermal progenitor cells. Representative FACS analysis results of mesenchymal stem cell markers of primary keratinocyte (CTL), or epidermal progenitor cells (EPCs) at passage 4. Human adipose mesenchymal stem cell (MSC) were used as positive control Figure 4 Skin irritation test using skin equivalents model generated by epidermal progenitor cells. (A) Representative images depicting H&E staining results of 3D skin models generated by using epidermal progenitor like (EPCs) after treated non‐irritant (Diethylphthalate) and irritant (Tetrachloroethylene) chemicals. 5% SDS was used as positive control for irritant chemical. (B) Cell viability of cell from 3D skin models treated non‐irritant and irritant chemicals. All data are shown as the mean ± SEM. *p < 0.05, compared to CTL via unpaired Student’s t‐test. Scale bar, 200 μm Figure 5 Effect of integrin beta 1 knockdown on the proliferation of epidermal progenitor cells. (A) FACS analysis of proliferative EPCs subjected to transfection with siCTL and siITGB1 at 300 pM or 1 nM of concentration, respectively. (B) Quantitative RT‐PCR analysis of proliferation and epidermal stem cell‐related markers in cells subjected to transduction with siCTL and siITGB1 at 300 pM or 1 nM of concentration, respectively. (C) Western blotting analysis for prolife [file CPR-55-e13284-s001.zip › Supplemental information - final clean version.docx]

The proliferative and multipotent epidermal progenitor cells for human skin reconstruction *in vitro* and *in vivo*

Jung Hwa Lim^1#^, Dae Hun Kim^1,2#^, Kyung Hee Noh^1^, Cho-Rok Jung^1,2*^, Hyun Mi Kang^1*^

.

^1^ Korea Research Institute of Bioscience and Biotechnology (KRIBB), 125 Gwahak-ro, Daejeon, Republic of Korea

^2^ Department of Functional Genomics, Korea University of Science and Technology (UST), 217 Gajeong-ro, Daejeon, Republic of Korea

^#^ These authors contributed equally to this work.

Correspondence and requests for materials should be addressed to Cho-Rok Jung ([crjung@kribb.re.kr](mailto:crjung@kribb.re.kr)) or Hyun Mi Kang ([hmkang@kribb.re.kr](mailto:hmkang@kribb.re.kr)).

**Supplemental information**

**Materials and Methods**

**Antibodies and reagents**

ITGB1 (IF, Abcam, #ab24693), cytokeratin 5 (IF, CST, #25807), Laminin 5 (IF, Abcam, #ab78286), TJP1 (IF, Novus biologicals, #NBP1-85046), CD11b (IF, BD Bioscience, #564454), cytokeratin 10 (IF, Santa Cruz Biotechnology, #sc53252), cytokeratin 14 (IF, Santa Cruz Biotechnology, #sc53253), involucrin (IF, Santa Cruz Biotechnology, #sc21748), filaggrin (IF, Santa Cruz Biotechnology , #sc66192), p63 (IF, Abcam, #ab111683), YAP1(IF, CST #14074S), aSMA (ICC, Abcam, #ab5694), N-cadherin (ICC, Santa Cruz Biotechnology , #sc59987), PCNA (IHC, Santa Cruz Biotechnology , #sc56), HuNuceloi (IHC, Abcam, #ab190710), muse Ki67 proliferation kit (Merck Millipore, Germany, #MCH100114-1), hMSC marker antibody panel (R&D, #SC017), dexamethasone (Sigma), 3-isobutyl-1-methylxanthine (Sigma), human insulin (Sigma), indomethacin (Sigma), b-glycerol phosphate (Sigma), ascorbic acid-2-phosphate (Sigma), sodium pyruvate (Gibco), transforming growth factor b3 (PeproTech) Insulin-Transferrin-Selenium (ITS, Gibco), bovine serum albumin (BSA, sigma), KGM-gold (Lonza), RPMI-1640 (Hyclone), DMEM-high glucose (Gibco), hydrocortisone (Sigma), Matrigel (Corning), fibronectin (Sigma), bovine collagen type I (Gibco), crystal violet (Sigma), Oil red O (Sigma), Alcian blue (Abcam), trichrome staining kit (Abcam).

**Colony-forming unit assay**

Primary keratinocytes or EPCs (1 × 10^3^ cells) were plated onto six-well culture plates. On day 10, cells were fixed with methanol and stained using crystal violet staining solution (5 min at room temperature). After washing and mounting, colonies between 1 and 8 mm in diameter (more than 20 cells) were counted.

**Quantitative real-time PCR**

RNA was isolated using the RNAeasy Mini kit; 1 μg was reverse transcribed using the cDNA archival kit (Life Technologies, Gaithersburg, MD), and qPCR was performed according to the manufacturers’ instructions (Applied Biosystems, Waltham, MA, USA, and Agilent Technologies) using SYBRGreen Master Mix. The data were normalized and analyzed using the ΔΔCT method. Primers used are listed in Supplementary Table I.

**Immunofluorescence and immunocytochemical analysis**

Cells were fixed with 4% paraformaldehyde and incubated in 2% goat or horse serum with 0.2% fish skin gelatin at room temperature for 1 h to block nonspecific binding. Cells were incubated with primary antibodies overnight at 4°C, and then secondary antibodies at room temperature for 1 h. Nuclei were counterstained with 4',6-diamidino-2-phenylindole.

**Flow cytometry**

Primary keratinocytes EPCs were harvested by trypsinization, washed, and resuspended in PBS supplemental with 2% FBS. Fluorescein conjugate primary antibody incubation was applied for 30 mins at 4°C and rinsed and resuspended in PBS. All data acquisition was performed on a FACS Aria or FACSCalibur flow cytometer (BD Biosciences) with CellQuest Pro software.

For MUSE analysis, cells treated drugs suspended 100 μl of serum free DMEM/F12 were transferred in suspension to a new tube and incubated with 100 μl of Muse Ki67 proliferation kit (Millipore) for 20 minutes at room temperature. The proliferation of cells was determined by Muse Cell Analyzer (Millipore) and the statistics were shown the percentages of the cells represented by population percentage in the stained cells.

**Skin irritant test**

For the irritant test, 30 μL of liquid materials were dispensed directly on the tissue surface and sterile forceps were used to tilt the insert and gently spread the liquid. After the materials were applied, the plates were incubated at 37°C and 5% CO_2_. After 45 min, the tissues were rinsed with D-PBS. Tissues were post-incubated for 42 h and then the entire medium was removed. Tissues were blotted and transferred to a 24-well plate that contained MTT (0.3 mg/mL) and incubated for 3 h at 37°C and 5% CO2. Next, tissues were rinsed with DPBS again and transferred to a new 6-well plate, prefilled with 2 mL of isopropanol. Formazan extraction was performed at room temperature for 2 h and 200 μL of formazan extract, per tissue was transferred to a 96 well plate. Optical density (OD) was measured at 570 nm using isopropanol as a blank.

**Scratch wound closure assay**

Primary keratinocytes or EPCs were seeded within 24-well plates, at 5x10^4^ cells/cm^2^, and allowed to incubate for a 24-hour period in KGM media to form a confluent monolayer. The KGM media was removed, and the confluent cell sheet was wounded through scratching the culture well surface with a 100-µL pipette tip. Cell migration was monitored by using the microscope over the 16-hour incubation.

**Tube formation assay**

Matrigel (12.5 mg/ml) was thawed at 4℃, and 10 μl was quickly added to each well of a 15-well μ-slide angiogenesis plate (Ibidi, USA) and then allowed to solidify for 30 min at 37℃. GFP-HUVEC cells (5x10^3^ cells) were subjected to the conditioned medium (CM-CTL) or EPCs conditioned medium (CM-EPCs) and added to each well and incubated for 6 h at 37℃. Experiments were performed in triplicate and repeated at least twice. With use of a fluorescence microscope (Olympus, Tokyo, Japan), five images per well were taken at ×100 magnification. The number of nodes (defined as at least three cells that formed a single point) per image was quantified.

**Angiogenesis antibody arrays**

The relative levels of human angiogenesis related proteins in EPCs were measured using a human angiogenesis antibody array kit (R&D systems). Control cells and EPCs were plated in the well of a six-well plate with 2 ml of KBM medium. After 48 h, the conditioned medium was collected and used for human angiogenesis antibody array analysis. The procedure was followed by the manufacturer’s instructions. Proteins were visualized using the ECL substrate kit and the results were analyzed with the Image J software.

**Supplemental figure 1. Expression of integrin beta 1 profiles in the primary keratinocytes.**

a. FACS analysis for skin stem cell marker of primary keratinocytes at passage 3. b. FACS analysis and sorting the integrin beta 1 (ITGB1) expressing cells in the three different primary keratinocytes at passage 3.

**Supplemental figure 2. Expression of skin stem cell markers of epidermal progenitor cells**

a. Representative immunostaining images of skin stem cell marker expression of primary keratinocyte (CTL) or epidermal progenitor cells (EPCs) at passage 5. b and c. FACS analysis for skin stem cell marker of EPSCs at passage 5. Scale bar, 50 μm.

**Supplemental figure 3. Expression of mesenchymal stem cell markers of epidermal progenitor cells**

Representative FACS analysis results of mesenchymal stem cell markers of primary keratinocyte (CTL), or epidermal progenitor cells (EPCs) at passage 4. Human adipose mesenchymal stem cell (MSC) were used as positive control.

**Supplemental figure 4. Skin irritation test using skin equivalents model generated by epidermal progenitor cells**

a. Representative images depicting H&E staining results of 3D skin models generated by using epidermal progenitor like (EPCs) after treated non-irritant (Diethylphthalate) and irritant (Tetrachloroethylene) chemicals. 5% SDS was used as positive control for irritant chemical. b. Cell viability of cell from 3D skin models treated non-irritant and irritant chemicals. All data are shown as the mean ± SEM. **p*<0.05, compared to CTL via unpaired Student’s *t*-test. Scale bar, 200 μm.

**Supplemental figure 5. Effect of integrin beta 1 knockdown on the proliferation of epidermal progenitor cells**a. FACS analysis of proliferative EPCs subjected to transfection with siCTL and siITGB1 at 300pM or 1nM of concentration, respectively. b. Quantitative RT-PCR analysis of proliferation and epidermal stem cell-related markers in cells subjected to transduction with siCTL and siITGB1 at 300pM or 1nM of concentration, respectively. c. Western blotting analysis for proliferation and epidermal stem cell-related markers in cells subjected to transduction with siCTL and siITGB1 at 300pM or 1nM of concentration, respectively. d. FACS analysis of proliferative EPCs subjected to treated with PBS (CTL) and ITGB1 blocking antibody at 1ug/ml or 5ug/ml of concentration, respectively.

**Supplemental figure 6. *In vivo* wound healing effects of epidermal progenitor cells**

a. Mice skin at 10 days post wound. Mice were randomly divided into four groups; CTL cell-injected, epidermal progenitor cell (EPCs)-injected, EPCs-transduced shCTL-injected and EPCs-transduced shITGB1-injected. b. Area percentage of wound healing area in mouse subjected to injection using different cells for 10 days post wound. All data are shown as the mean ± SEM. **p*<0.05, compared to CTL via unpaired Student’s *t*-test.

**Supplemental figure 7. *In vivo* function analysis of repaired skin treated with epidermal progenitor cells**

Immunostaining for the epidermal polarity (CK14), the barrier function (tight junction protein-1, TJP1) of the epidermis, a major link between the epidermis and dermis (laminin 5), and pro-inflammation response (CD11b) in the dermis of the mice injected with different cells: CTL cell-injected (I), epidermal progenitor cell (EPCs)-injected (II), EPCs-transduced shCTL-injected (III) and EPCs-transduced shITGB1-injected (IV). Normal skin tissues were used as a positive control. Scale bar, 50 μm.

**Supplemental Table I. Sequences of oligonucleotide primers used for qPCR.**
