# Supplementary material for: The proliferative and multipotent epidermal progenitor cells for human skin reconstruction in vitro and in vivo
Source: Cell Prolif. 2022 Jun 20;55(9):e13284. doi: 10.1111/cpr.13284 (PMC9436902; doi:10.1111/cpr.13284)
Supplement: Supplementary file 1 — Appendix S1 Figure 1 Expression of integrin beta 1 profiles in the primary keratinocytes. (A) FACS analysis for skin stem cell marker of primary keratinocytes at passage 3. (B) FACS analysis and sorting the integrin beta 1 (ITGB1) expressing cells in the three different primary keratinocytes at passage 3 Figure 2 Expression of skin stem cell markers of epidermal progenitor cells. (A) Representative immunostaining images of skin stem cell marker expression of primary keratinocyte (CTL) or epidermal progenitor cells (EPCs) at passage 5. (B) and (C). FACS analysis for skin stem cell marker of EPCs at passage 5. Scale bar, 50 μm Figure 3 Expression of mesenchymal stem cell markers of epidermal progenitor cells. Representative FACS analysis results of mesenchymal stem cell markers of primary keratinocyte (CTL), or epidermal progenitor cells (EPCs) at passage 4. Human adipose mesenchymal stem cell (MSC) were used as positive control Figure 4 Skin irritation test using skin equivalents model generated by epidermal progenitor cells. (A) Representative images depicting H&E staining results of 3D skin models generated by using epidermal progenitor like (EPCs) after treated non‐irritant (Diethylphthalate) and irritant (Tetrachloroethylene) chemicals. 5% SDS was used as positive control for irritant chemical. (B) Cell viability of cell from 3D skin models treated non‐irritant and irritant chemicals. All data are shown as the mean ± SEM. *p < 0.05, compared to CTL via unpaired Student’s t‐test. Scale bar, 200 μm Figure 5 Effect of integrin beta 1 knockdown on the proliferation of epidermal progenitor cells. (A) FACS analysis of proliferative EPCs subjected to transfection with siCTL and siITGB1 at 300 pM or 1 nM of concentration, respectively. (B) Quantitative RT‐PCR analysis of proliferation and epidermal stem cell‐related markers in cells subjected to transduction with siCTL and siITGB1 at 300 pM or 1 nM of concentration, respectively. (C) Western blotting analysis for prolife [file CPR-55-e13284-s001.zip › EPCs supplemental figure - revision.pptx]

## Slide 1
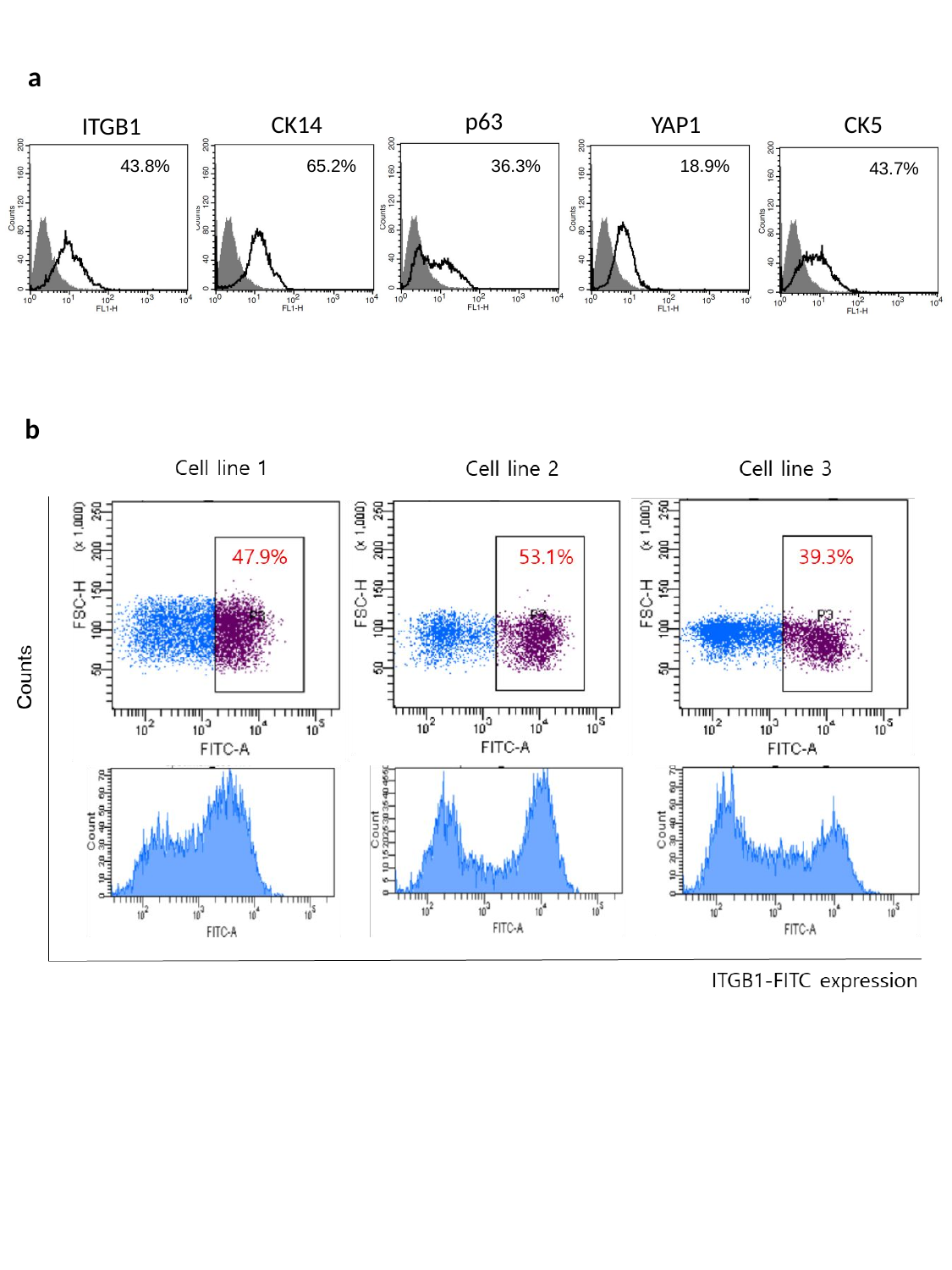

a
p63
YAP1
CK14
ITGB1
65.2%
18.9%
43.8%
36.3%
CK5
43.7%
b

## Slide 2
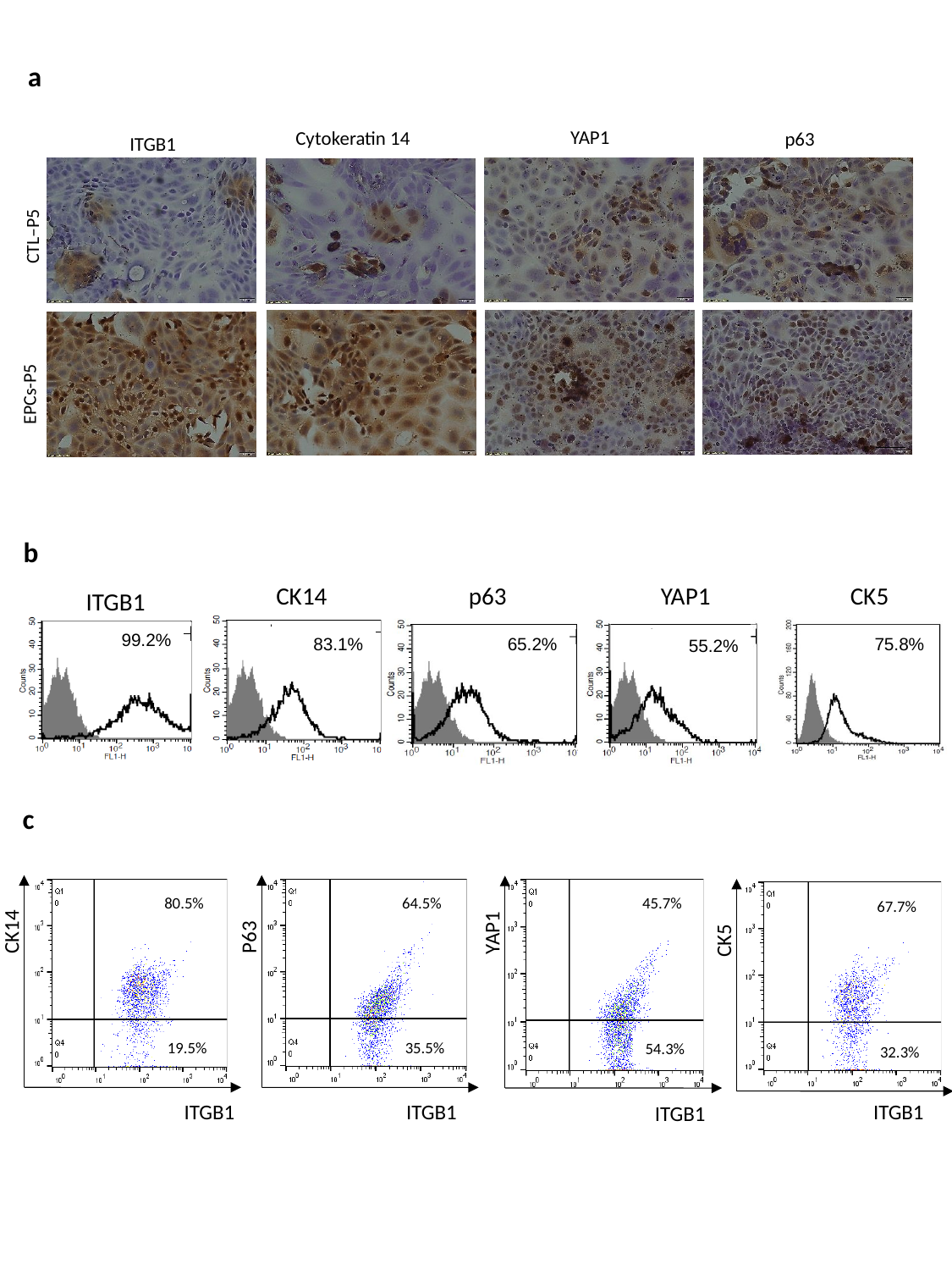

a
Cytokeratin 14
CTL–P5
YAP1
ITGB1
p63
EPCs-P5
b
YAP1
CK14
p63
ITGB1
83.1%
55.2%
99.2%
65.2%
CK5
75.8%
c
80.5%
CK14
19.5%
ITGB1
64.5%
P63
35.5%
45.7%
YAP1
54.3%
67.7%
CK5
32.3%
ITGB1
ITGB1
ITGB1

## Slide 3
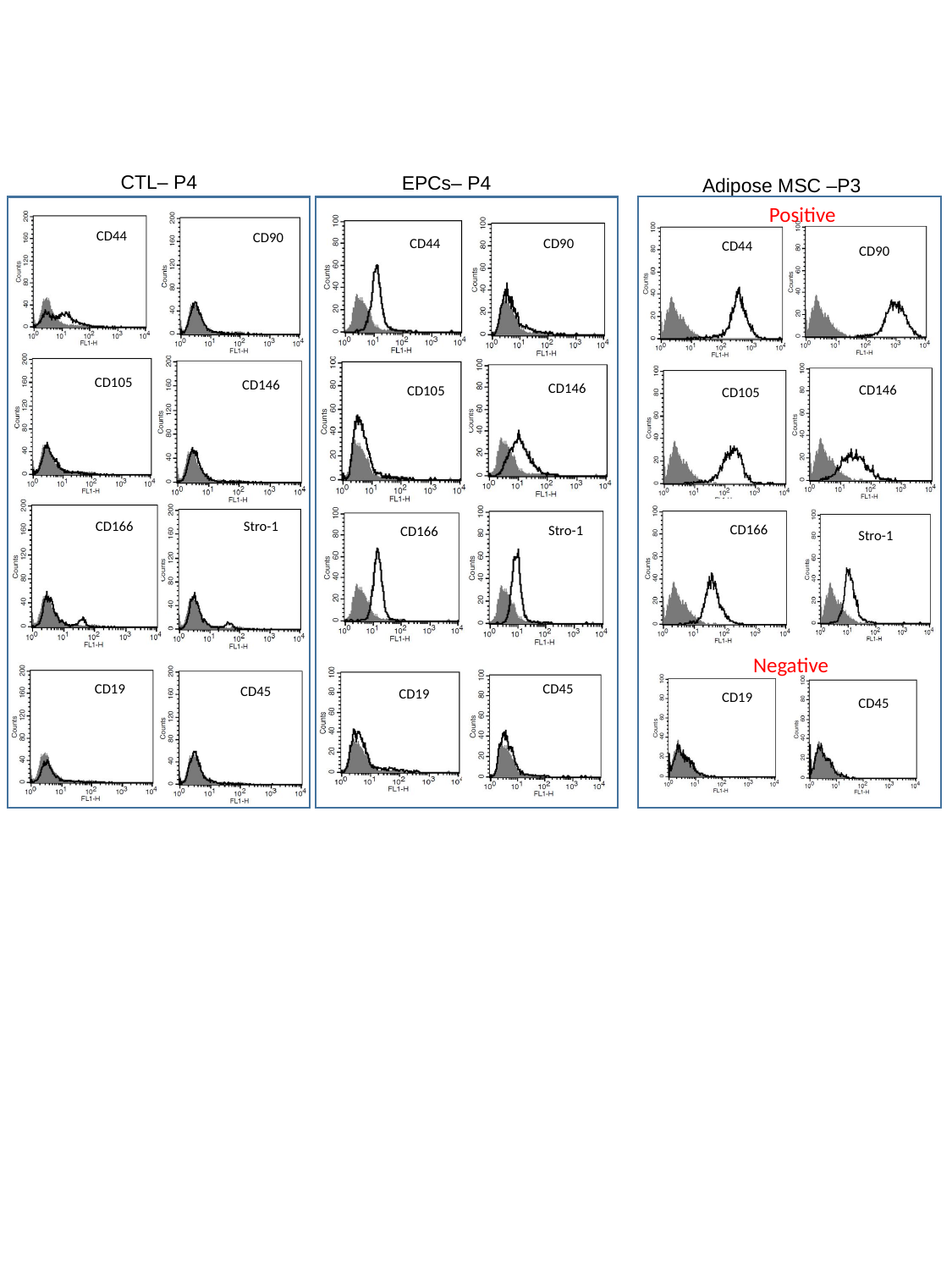

CTL– P4
EPCs– P4
Adipose MSC –P3
Positive
CD44
CD90
CD44
CD90
CD44
CD90
CD105
CD146
CD146
CD146
CD105
CD105
CD166
Stro-1
CD166
Stro-1
CD166
Stro-1
Negative
CD19
CD45
CD45
CD19
CD19
CD45

## Slide 4
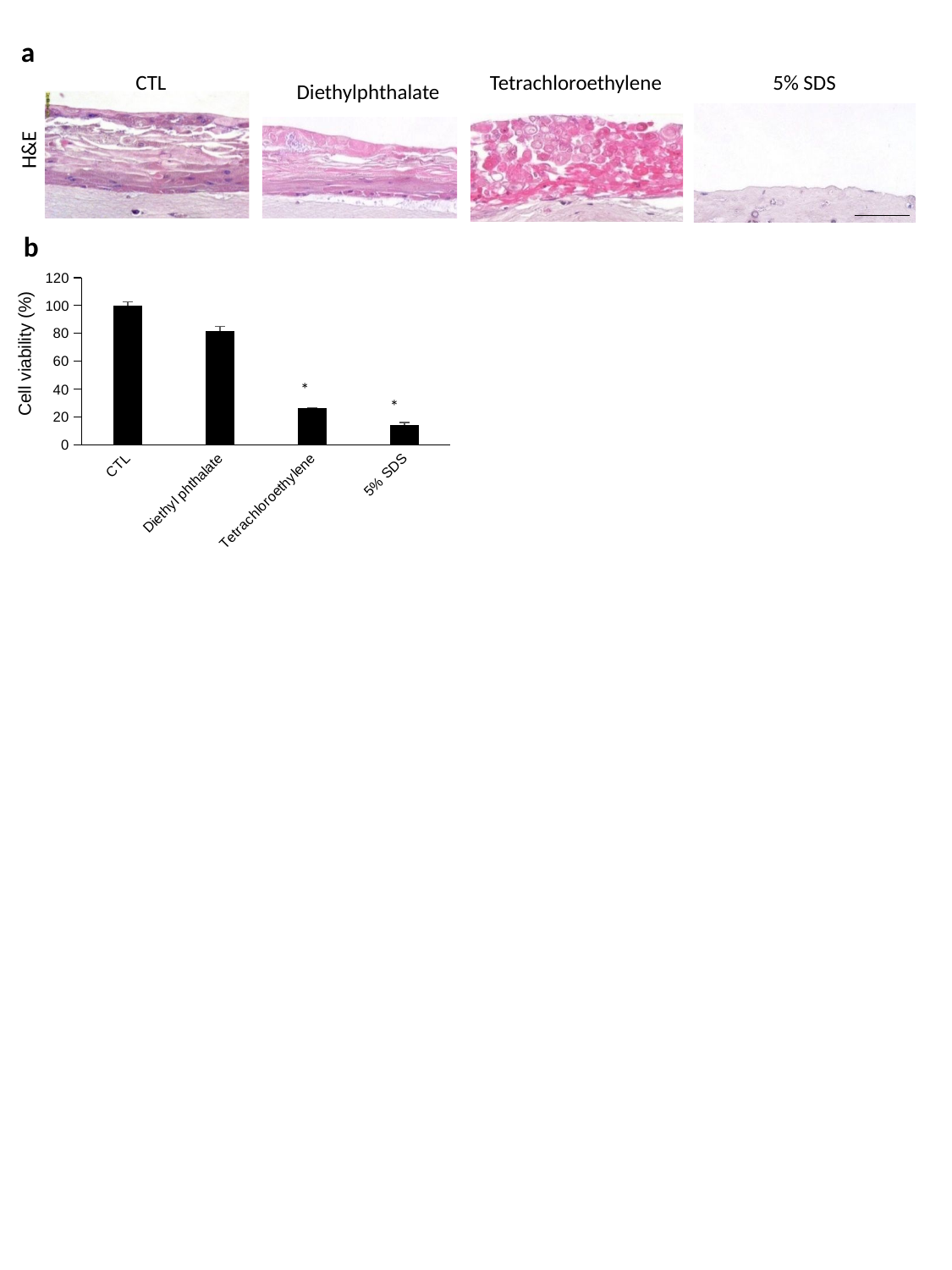

a
Diethylphthalate
CTL
Tetrachloroethylene
5% SDS
H&E
b
Cell viability (%)
### Chart
| Category | |
|---|---|
| CTL | 100.0 |
| Diethyl phthalate | 81.87393156595121 |
| Tetrachloroethylene | 26.185113830624374 |
| 5% SDS | 14.522562613627112 |*
*

## Slide 5
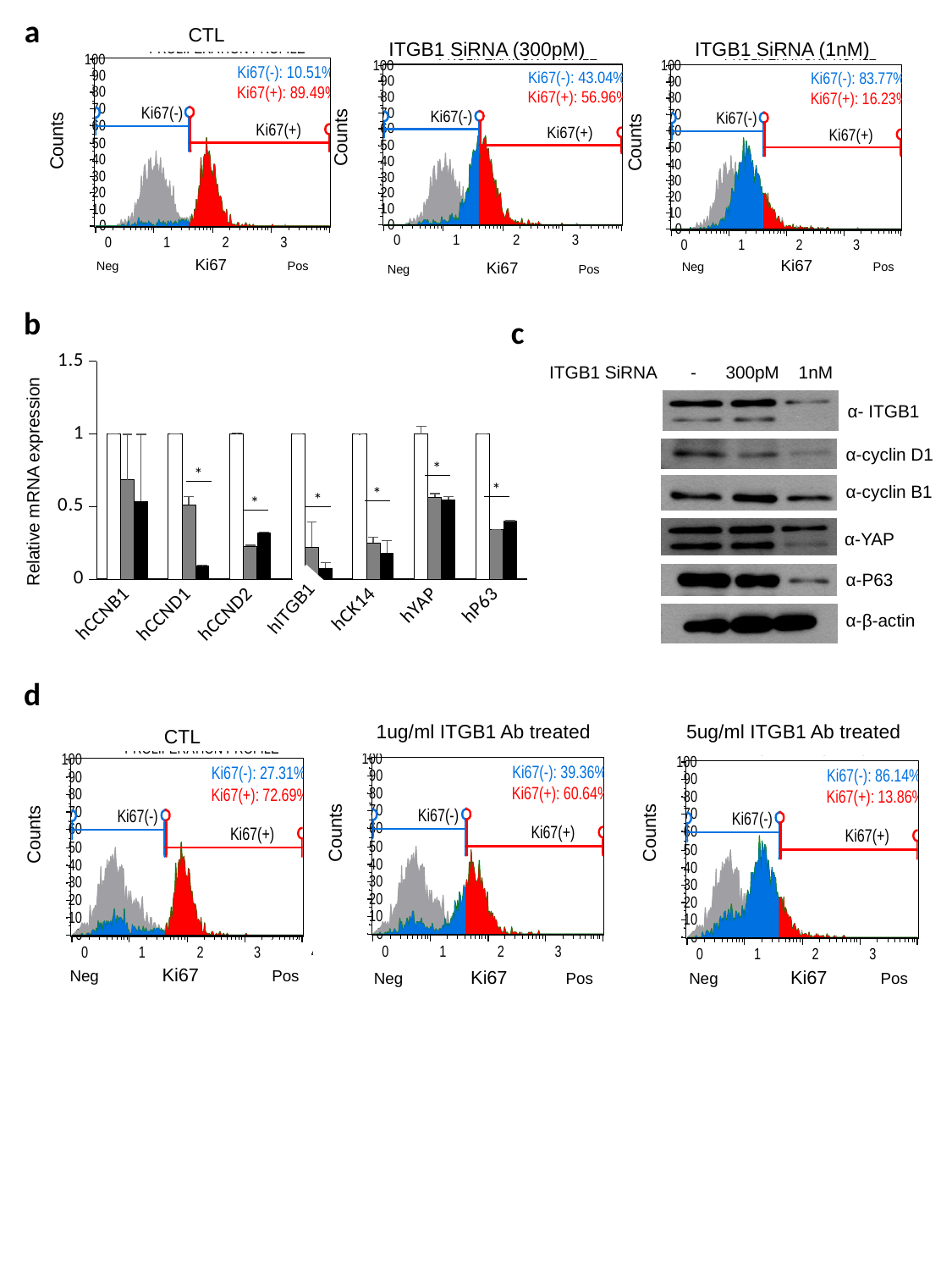

a
CTL
Counts
Neg Ki67 Pos
ITGB1 SiRNA (300pM)
Counts
Neg Ki67 Pos
ITGB1 SiRNA (1nM)
Counts
Neg Ki67 Pos
b
c
Relative mRNA expression
### Chart
| Category | | Ab1μg | Ab5μg |
|---|---|---|---|
| hCCNB1 | 1.0 | 0.6847539031436732 | 0.5365982162213349 |
| hCCND1 | 1.0 | 0.5125766455900055 | 0.09465044498350832 |
| hCCND2 | 1.0 | 0.2279298414596746 | 0.32380164384364907 |
| hCD29 | 1.0 | 0.22135356744599505 | 0.07463668381602191 |
| hCK14 | 1.0 | 0.2498753753730576 | 0.17950854926202012 |
| hYAP | 1.0 | 0.5665716749154384 | 0.5459477519379455 |
| hP63 | 1.0 | 0.34079369360411776 | 0.4027245017650384 |ITGB1 SiRNA - 300pM 1nM
α- ITGB1
α-cyclin D1
α-cyclin B1
α-β-actin
α-YAP
α-P63
*
*
*
*
*
*
hITGB1
d
1ug/ml ITGB1 Ab treated
Counts
Neg Ki67 Pos
5ug/ml ITGB1 Ab treated
Counts
Neg Ki67 Pos
CTL
Counts
Neg Ki67 Pos

## Slide 6
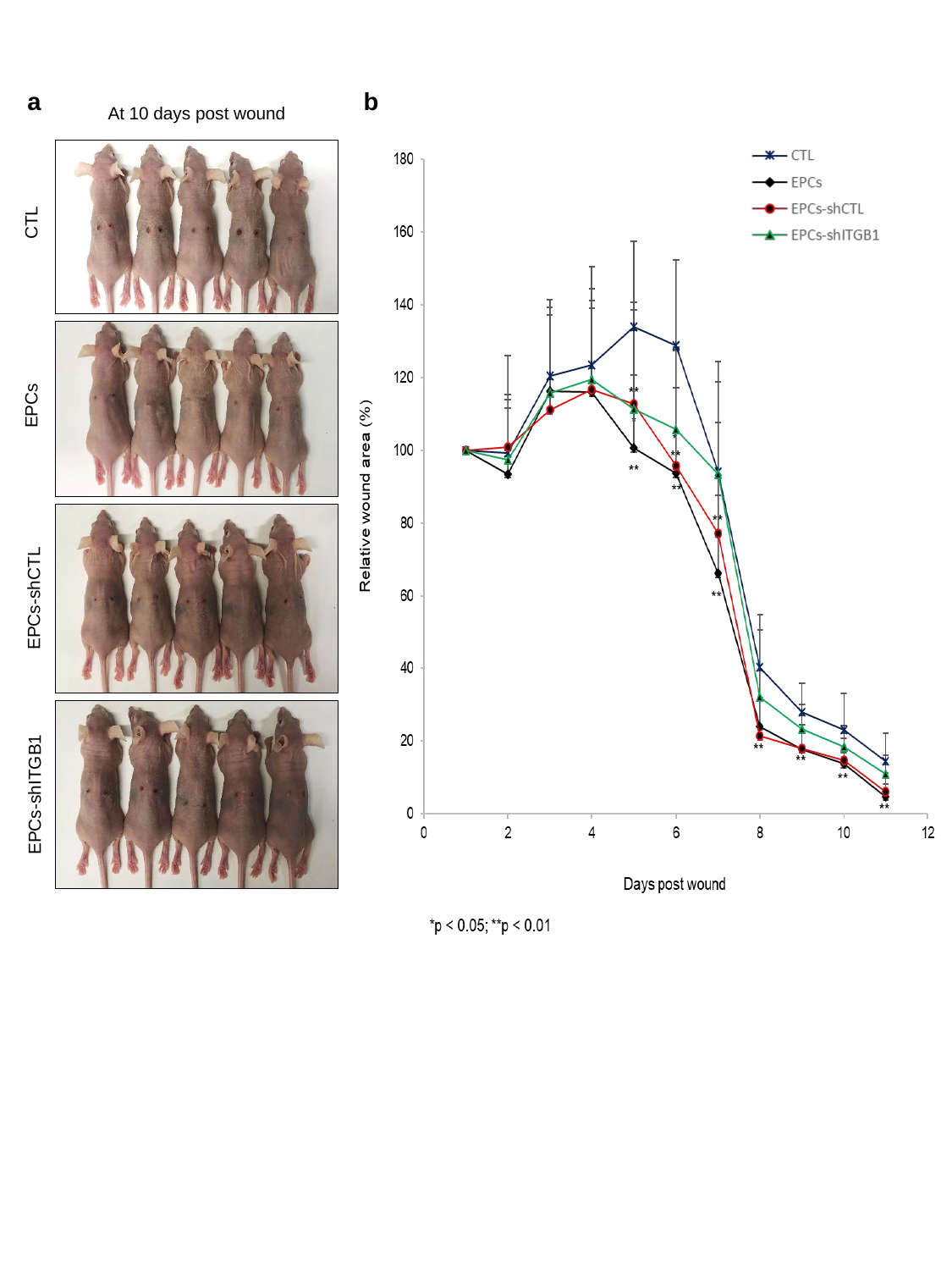

b
a
At 10 days post wound
CTL
EPCs
EPCs-shCTL
EPCs-shITGB1

## Slide 7
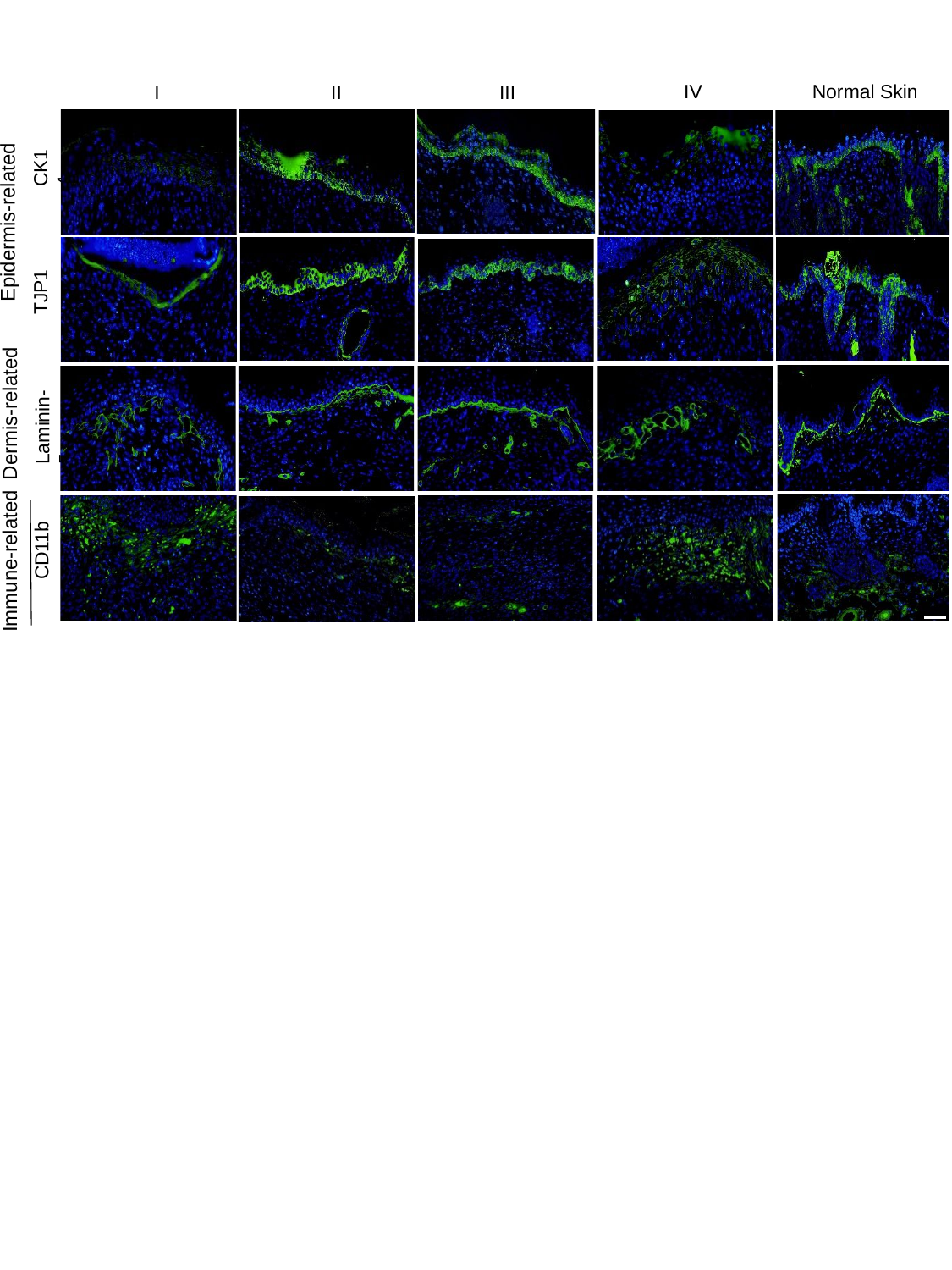

IV
Normal Skin
I
II
III
CK14
TJP1
Laminin-5
CD11b
Epidermis-related
Dermis-related
Immune-related
